# A PIM-1 Kinase Inhibitor Docking Optimization Study Based on Logistic Regression Models and Interaction Analysis

**DOI:** 10.3390/life13081635

**Published:** 2023-07-27

**Authors:** George Nicolae Daniel Ion, George Mihai Nitulescu, Dragos Paul Mihai

**Affiliations:** Faculty of Pharmacy, “Carol Davila” University of Medicine and Pharmacy, Traian Vuia 6, 020956 Bucharest, Romania; daniel.ion@drd.umfcd.ro (G.N.D.I.); dragos_mihai@umfcd.ro (D.P.M.)

**Keywords:** PIM-1 kinase, PIM-1 inhibitors, protein kinase inhibitors, virtual screening, predictive score, amino acid interactions, logistic regression, data clustering

## Abstract

PIM-1 kinase is a serine-threonine phosphorylating enzyme with implications in multiple types of malignancies, including prostate, breast, and blood cancers. Developing better search methodologies for PIM-1 kinase inhibitors may be a good strategy to speed up the discovery of an oncological drug approved for targeting this specific kinase. Computer-aided screening methods are promising approaches for the discovery of novel therapeutics, although certain limitations should be addressed. A frequent omission that is encountered in molecular docking is the lack of proper implementation of scoring functions and algorithms on the post-docking results, which usually alters the outcome of the virtual screening. The current study suggests a method for post-processing docking results, expressed either as binding affinity or score, that considers different binding modes of known inhibitors to the studied targets while making use of in vitro data, where available. The docking protocol successfully discriminated between known PIM-1 kinase inhibitors and decoy molecules, although binding energies alone were not sufficient to ensure a successful prediction. Logistic regression models were trained to predict the probability of PIM-1 kinase inhibitory activity based on binding energies and the presence of interactions with identified key amino acid residues. The selected model showed 80.9% true positive and 81.4% true negative rates. The discussed approach can be further applied in large-scale molecular docking campaigns to increase hit discovery success rates.

## 1. Introduction

Oncological targets are nowadays one of the most studied topics in health sciences, in hope of better identifying and responding to most types of cancerous diseases existing worldwide [1]. One of the most studied classes of oncological targets is represented by protein kinases, as these enzymes regulate crucial processes in the tumoral cell cycle, proliferation, and drug-resistance mechanisms [2]. Such an interesting intracellular signaling enzyme emerging as a suitable drug target in recent years is PIM-1, a serine-threonine kinase encoded by the oncogene with the same name. Originally discovered as a proviral integration site for the Moloney murine leukemia virus, hence its name, PIM kinase has multiple implications in tumoral formation and growth, regulating processes such as cell survival and proliferation, cell cycle, and chemoresistance [3]. Its implications in several types of cancers, such as prostate cancer, breast cancer, and colorectal cancer, as well as acute myeloid leukemia and other hematologic malignancies, make it a promising oncologic drug target if its activity could be inhibited by small-molecule drugs [4]. Unfortunately, currently there are as yet no approved PIM-1 inhibitors as a therapy for these malignancies, even though various compounds have been commercialized as in vitro inhibitors for research use, many being implicated in preclinical studies [5].

The traditional path of drug discovery for such a target involves starting with computational studies, often leading to large virtual screening experiments on hundreds or thousands of compounds, many of which will most probably not succeed as approved drugs [6]. However, many biologists, chemists, or biochemists tend to not rely on the predictive power of virtual screening studies, keeping in mind that in silico studies only point the direction for subsequent, more expensive in vitro and in cellulo experiments. Although docking studies are an empirically validated method for the prediction of pharmacological activity, frequently being able to show a molecule’s potential to exhibit inhibitory activity over a certain drug target, they cannot explain with sufficient consistency the “real” inhibitory activity displayed later in enzymatic assays or cell culture experiments [7]. Usually, the proportionality between the predicted score or binding energy and in vitro IC_50_ or EC_50_ is not very strong. This aspect is, most of the time, related to the complex cellular mechanisms such as cell signaling and drug metabolism, which may in turn even affect the drug candidate’s bioavailability at the site of action, so they should be identified and taken into account when corroborating computational studies’ results with those of in vitro experiments [8]. 

However, one other impactful source of error may come from the docking study algorithm, as these types of experiments usually follow a general docking method used for most drug targets, which is then applied as such for screening different kinds of molecules against one specific target. Depending on the complexity of the docking method and software used, simulation results, frequently expressed as binding energy, binding affinity, or docking score can be more or less correlated with in vitro results such as IC_50_ or EC_50_, although molecular docking usually identifies several key interactions between a drug candidate and the targeted protein.

Our approach to virtual screening studies follows the principle that proteins with different structures require different methods of transformation and interpretation of the docking results, based on certain features, such as specific amino acids or protein regions. 

Following the same principle, several cheminformatics descriptors, known as interaction fingerprints, were introduced as early as 2004, when Deng et al. described the use of structural interaction fingerprints (SIFt) for clustering and characterization of kinase inhibitor complexes [9]. Since then, various methods have been developed and effectively utilized to convert interactions observed in 3D structural data into binary fingerprints for a wide range of applications [10]. These interaction fingerprints are usually binary vectors, or strings, coding in various ways interactions between the ligand and the amino acid residues of the targeted protein, estimated from the docking study [11].

The current study focuses on exploring different interactions and binding modes observed through molecular docking studies between a set of known, in vitro-tested inhibitors and the target enzyme, PIM-1 kinase.

For this purpose, we considered analyzing binding modalities of different known PIM-1 kinase inhibitors, comparing them to a decoy set of compounds not specifically targeting the protein kinase, by looking at each interaction between our sets of molecules and all of the residues involved in every docking simulation for those compounds. Our hypothesis suggested that some of the analyzed compounds would better fit into a predictive pattern than others, based on their chemical structure. As such, adjusting the docking result, expressed as binding energy, by a certain correction factor would yield a better prediction when estimating potential inhibitory activity than the actual binding energy displayed by the docking software.

## 2. Materials and Methods

### 2.1. Creating the Compounds Dataset

The selection of compounds to be studied started from a dataset downloaded from the ChEMBL database [12,13], consisting of 3067 IC_50_ activities testing inhibition of the human PIM-1 kinase target. Downloaded data were managed with Microsoft Excel 2019 and OSIRIS DataWarrior 5.5.0 software [14]. Data preparation consisted of filtering for duplicates, inexact IC_50_ values, and selection of IC_50_ results expressed only in µM units (or conversion where possible), excluding inorganic combinations and compounds containing metal atoms. The cleaned dataset resulted in 2551 compounds, representing the final inhibitor test set. The chemical structures and data regarding tested inhibitors are available in Supplementary Materials, Table S1.

Another set of randomly selected compounds, termed the “decoy” set, was generated for comparison of binding interactions. The ChEMBL database was downloaded as a 6-part .sdf file, then each subset was filtered for certain chemical descriptor values matching the inhibitor set for physicochemical similarity (filter properties used are detailed in Table 1). Starting from approximately 2.4 million compounds, a set of approximately 16,000 compounds was selected. After cleaning the set for compounds with no metal atoms, an equal number of compounds was randomly selected from each subset using the Diverse Cluster function of Data Warrior software (Version 5.5.0), in order to obtain structural diversity in the decoy set. The final decoy set had a 1:1 ratio to the PIM-1 kinase inhibitors set. 

### 2.2. Molecular Docking

Molecular docking simulations were performed to predict the binding affinities and molecular interactions for both PIM-1 kinase inhibitor and decoy sets. Crystal structure of human PIM-1 kinase in complex with a [1,2,4]triazolo [4,3-b]pyridazine-based inhibitor was retrieved from the RCSB PDB database (PDB ID: 3 bgq, 2.00 Å resolution [15]). The target structure was prepared for docking with YASARA Structure [16] by adding missing hydrogens according to the physiological pH (7.4), optimizing the hydrogen bond network, correcting structural errors, and energy minimization of the protein–ligand complex using the NOVA2 forcefield. Moreover, all water molecules were removed, except for one structural water that forms a hydrogen bond network between the ligand and Glu89 within the αC-helix. The co-crystallized ligand was removed and re-docked in the binding pocket for validating the docking protocol. The predicted pose of the experimental ligand was superposed on the original conformation to calculate the root mean square deviation (RMSD).

Ligand virtual libraries containing structures of PIM-1 inhibitors and decoys were prepared with DataWarrior by generating the 3D structures, energy minimization using the MMFF94s+ forcefield, and protonation according to the physiological pH. AutoDock Vina v.1.1.2 [17] and AutoDock4 (Lamarckian Genetic Algorithm) [18] algorithms were used within YASARA for the docking experiments, with a search space of 25 × 25 × 25 Å that was centered around the co-crystallized ligand within the active site. A total of 20 docking runs were executed for each ligand. The two experiments were performed in order to establish which algorithm is more suitable for predicting the affinity and binding pose of PIM-1 inhibitors.

The docking results were retrieved as binding energies (ΔG, kcal/mol) and interacting residues for the best binding pose of each ligand. The docking poses and predicted molecular interactions between the target protein and ligands were analyzed using BIOVIA Discovery Studio Visualizer (BIOVIA, Discovery Studio Visualizer, Version 17.2.0, Dassault Systèmes, 2016, San Diego, CA, USA).

### 2.3. Ligand Interactions Analysis

Docking results for both inhibitor and decoy sets were exported and interpreted in Microsoft Excel and SPSS Statistics 26.0 software [19]. Bulk results, expressed in .txt format, consisted of binding energy (in kcal/mol) and the name of every interacting amino acid residue for each ligand. For further analysis, interactions of each docked ligand with every participating amino acid residue were coded as a binary result for each of the interacting residues of the protein, with 1 marking the presence, and 0 representing an absence of interaction with that residue, respectively.

### 2.4. Logistic Regression

The binary logistic regression method was used in SPSS to optimize the results generated after molecular docking. A set of new variables was introduced as an indicator of a strong or weak inhibitor of PIM-1 kinase. The threshold for separating the two classes was represented by the negative log of the IC_50_ value expressed in molar units (pIC_50_), based on the following formula:

Class N = 0 if pIC_50_ < N,

Class N = 1 if pIC_50_ ≥ N,

where N ∈ (4, 7).

The binary logistic regression was performed using as a dependent variable each class N variable. The model was constructed by selecting the covariates using the forward Wald method.

### 2.5. Data Clustering by Residue Interaction

In order to obtain more information about the different binding modalities of the ligands with the target protein, we performed a classification analysis based on a two-step clustering method, suitable for datasets such as the current one with a large number of binary data. The clustering analysis was perfomed in order to identify groups of compounds that bind similarly to the protein based on their interactions with amino acid residues. This approach helps uncover similarities and differences in the binding profiles of certain ligands and can aid in understanding structure–activity relationships and identifying compounds with similar interaction patterns.

Using SPSS 26.0.0.0 software, we conducted a two-step clustering analysis of the interaction results on the dataset of 5080 cases (inhibitor and decoy molecules) with the 50 binary asymmetrical ordinal variables (presence vs. absence of interaction with every amino acid residue). After randomly arranging the cases in the data table for minimization of order effects, clustering classification analysis was performed using Schwartz’s Bayesian information criterion (BIC) and log-likelihood method for measuring the distance between cases. The 50 variables representing interacting residues were set as categorical variables and the maximum number of clusters possible was determined automatically and set to 15.

In other words, for this analysis, every case (compound) was treated like a binary vector coding interactions with every participating amino acid residue of the protein. First, distances between each case were calculated based on similarity and dissimilarity between vectors, pairwise. Here, the log-likelihood method was used to measure the dissimilarity between cases, as it is known to be suitable for categorical binary variables such as the presence or absence of interactions with a residue. Afterward, the hierarchical clustering algorithm selected for this analysis started from individual clusters and iteratively merged two of the closest clusters. At each step, it identified the two closest clusters based on the log-likelihood distances. The choice for merging the linkage criteria was calculated using Ward’s method, which minimizes the increase in variance when merging cases. Then, the dissimilarity matrix was recalculated for the newly-formed cluster and the remaining clusters based on the same log-likelihood distances. These steps were repeated until all cases were merged into the number of clusters calculated. In this case, the Bayesian information criterion, a statistical criterion for clustering analysis that balances the goodness of fit of the clustering model with its complexity, established the optimum number of clusters at 2. More information about the difference between clustering analysis methods can be found in dedicated statistical works [10].

### 2.6. Control Docking Study

As a final analysis, we performed a second screening study on the two groups of 2546 inhibitors and 2534 decoys in order to check the accuracy of the docking algorithm used. Therefore, using Yasara v.22.5.22 software, we performed the docking study again, with exactly the same settings as before, except for the docking algorithm used, which was this time selected as Autodock [18], instead of VINA, the only other computational approach available in Yasara docking software for calculating binding energy. Ligands and molecules were used as prepared for the first study, with the gridbox and all other preparative operations kept identical. We then performed correlation and regression analysis on the results, for comparison with docking energy values calculated by VINA.

## 3. Results

### 3.1. Datasets Generation

#### 3.1.1. PIM-1 Kinase Inhibitors

From our initial refined database of 2551, 5 compounds could not be properly docked due to unknown structural properties; therefore, the inhibitor set finally consisted of 2546 compounds tested for PIM-1 kinase activity. Descriptive statistics of the chemical descriptors for this set are displayed in Table 2.

#### 3.1.2. Decoy Set

After reuniting the 6 selected subsets of ChEMBL compounds and cleaning the dataset, the decoy set finally consisted of 2534 structurally diverse compounds. Descriptive statistics of the chemical descriptors for this set are displayed in Table 3.

### 3.2. Physicochemical Descriptor Analysis

**Figure 1:**
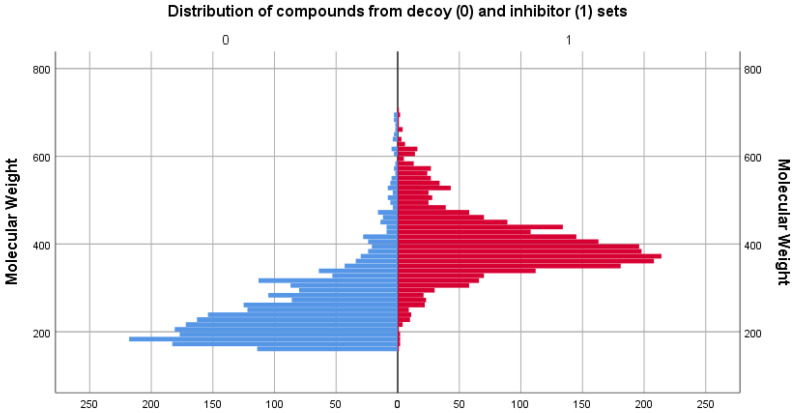
Comparison between the distribution of molecular weight values of the decoy (blue) and inhibitor (red) sets.

**Figure 2:**
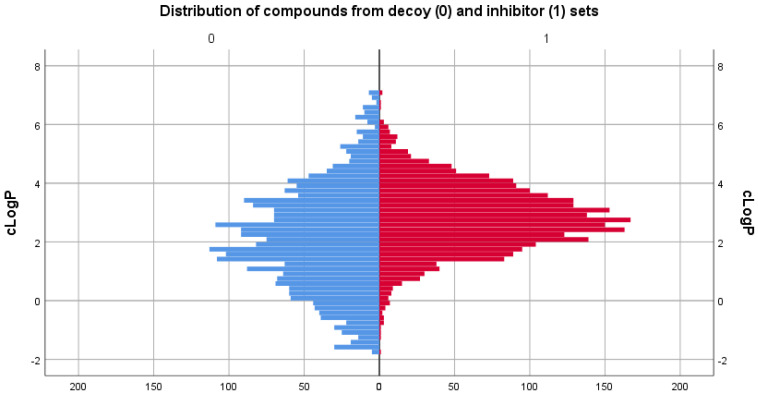
Comparison between the distribution of clogP values of the decoy (blue) and inhibitor (red) sets.

**Figure 3:**
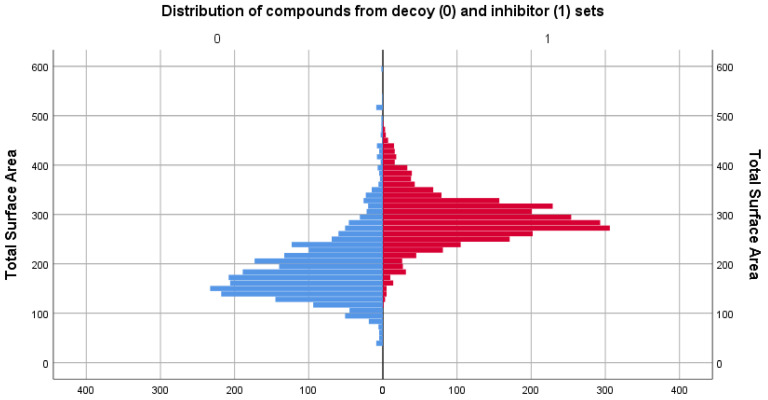
Comparison between the distribution of total surface area descriptor values of the decoy (blue) and inhibitor (red) sets.

**Figure 4:**
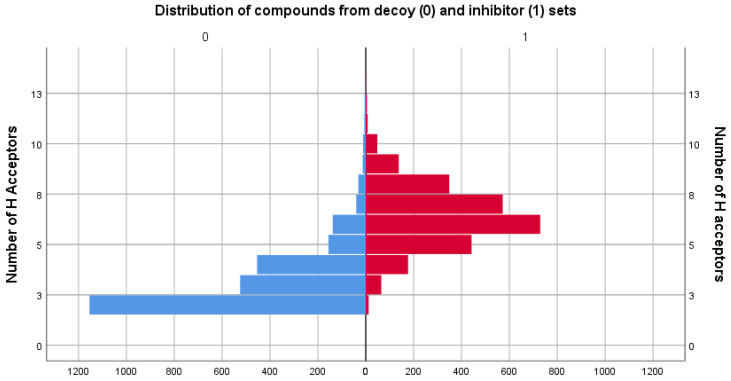
Comparison between the distribution of the number of H acceptors descriptor values of the decoy (blue) and inhibitor (red) sets.

**Figure 5:**
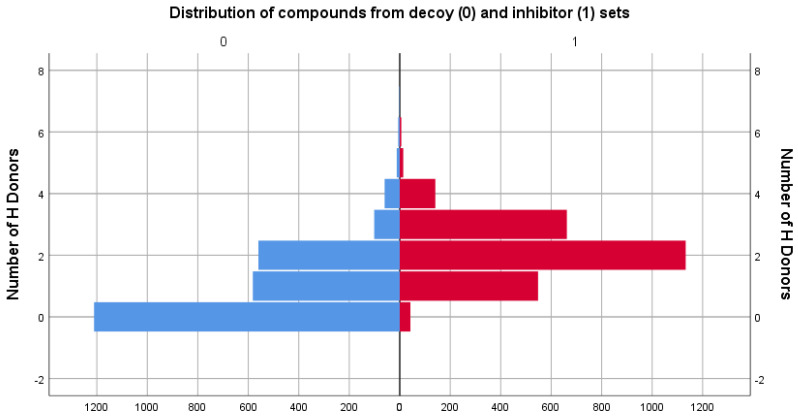
Comparison between the distribution of the number of H donors descriptor values of the decoy (blue) and inhibitor (red) sets.

As the two sets compared consisted of different types of molecules, the distributions of their chemical descriptors did not match exactly. However, by filtering the decoy set only for values between the range values of descriptors in Table 1, it was ensured that the untested molecules were similar in regard to physicochemical properties to those of the inhibitor set. For comparison, distribution graphs of these chemical descriptor values for the two sets are presented below, in Figure 1, Figure 2, Figure 3, Figure 4 and Figure 5.

Regarding the molecular weight descriptor, it can be observed that the inhibitor displays a slightly higher mean (401.34) compared with the decoy set (260.50), suggesting that the compounds in the inhibitor set tend to have larger molecular weights on average, as they were designed specifically to fill the binding pocket of the targeted protein.

In terms of octanol/water partition coefficient, the inhibitor set has a slightly higher mean cLogP value (2.831) compared with the decoy set (2.102), indicating that the compounds in the inhibitor set may have slightly higher hydrophobicity.

The inhibitor set has a higher mean surface area (290.523) compared with the decoy set (192.756), suggesting that the PIM-1 inhibitors set may have larger and more complex molecular structures.

In regard to hydrogen bond formation capability, the inhibitor set has a higher mean for both descriptors, HBA and HBD, compared with the decoy set, indicating a higher potential for hydrogen bonding interactions in the inhibitor set compounds.

As observable from the comparative analyses, the two sets do not share similar distributions of the previously mentioned physicochemical descriptor values. However, this aspect is to be expected, bearing in mind the more diverse chemical space represented by the decoy group, compared with the more specific, pocket-targeted molecules of the inhibitors group.

### 3.3. Molecular Docking

**Figure 6:**
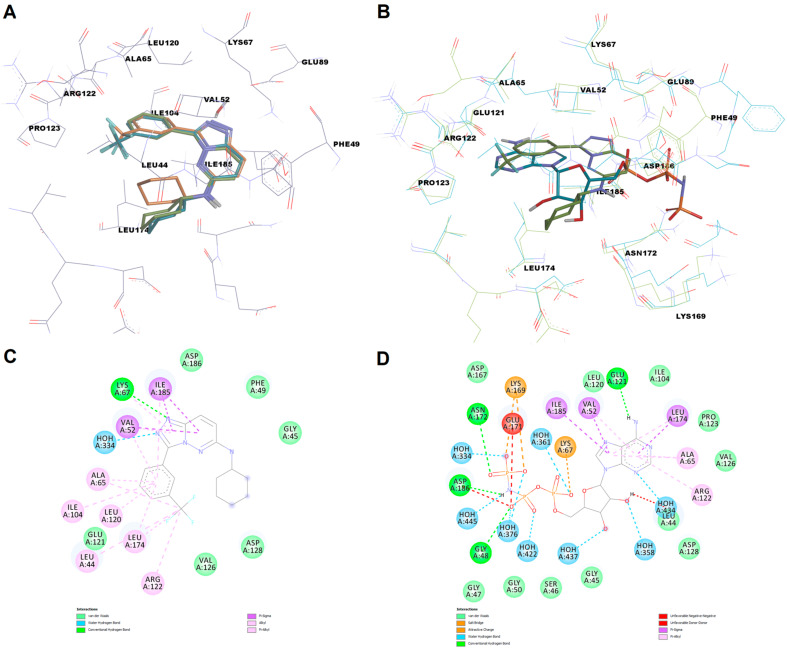
(**A**)—superposition of the predicted binding poses (AutoDock Vina—blue, AutoDock4—orange) of the co-crystallized triazolo-pyridazine inhibitor on the experimental conformation (green); (**B**)—superposition of the binding site conformation of the inhibitor-bound PIM-1 (blue) on the AMP-PNP-bound enzyme (green); (**C**)—2D diagram of molecular interactions between the co-crystallized inhibitor and PIM-1 kinase; (**D**)—2D diagram of molecular interactions between AMP-PNP and PIM-1 kinase.

The molecular docking protocols were first validated by superposing the predicted conformation of the co-crystallized ligand onto the experimentally determined pose. The calculated RMSD value after superposition was 0.2197 Å for AutoDock Vina, indicating good accuracy in predicting the correct binding pose (Figure 6A). On the other hand, the RMSD value after docking with AutoDock4 was 2.0193 Å, showing a less reliable binding pose prediction. In the case of AutoDock4, there was a high variation in the orientation of the cyclohexyl substructure, which engaged in two more interactions with pocket residues (Leu44 and Leu174), the predicted pose having also a higher torsion. Therefore, we considered that, in this specific case, AutoDock Vina was more suitable for correctly predicting the ligand conformation and interactions.

We further superposed the binding pocket of PIM-1 in complex with an inhibitor on the pocket conformation of the same kinase in complex with AMP-PNP (adenylyl-imidodiphosphate), a nonhydrolyzable ATP analog, after preparation in identical conditions (PDB ID: 1yxt [20]), to highlight the differences in amino acid residues orientation and the similarities between the two ligands regarding the interactions with the active site (Figure 6B). Interestingly, both PIM-1 structures have the αC_in_ architecture (a salt bridge can be formed between the charged Lys67 within the β3-strand and Glu89 within the αC-helix) and are in active DFG_in_ conformation, since Phe187 is packed under the αC-helix. The major difference between the two binding site conformations is the fact that in the AMP-PNP-bound protein, Phe49, is displaced from the cavity by the γ-phosphate moiety of AMP-PNP. In the protein-inhibitor complex, Phe49 adopts an orientation similar to that observed in the apo structure of PIM-1. The triazolo-pyridazine scaffold of the co-crystallized inhibitor binds to the active site in a similar manner to both the α-phosphate and adenine moieties of AMP-PNP, by forming electrostatic interactions with Lys67 (hydrogen bond vs. salt bridge), a water hydrogen bond with a conserved structural water molecule, and nonpolar pi-sigma interactions with a conserved valine (Val52) and Ile185. The trifluoromethyl-phenyl substructure also mimics some of the hydrophobic interactions between the adenine moiety and the enzyme (e.g., interactions with Ala65, Arg122, Leu174). Moreover, the cyclohexyl moiety occupies relatively the same space in the binding site as the ribose moiety and engages only in weak van der Waals interaction with the enzyme (Figure 6C,D).

**Figure 7:**
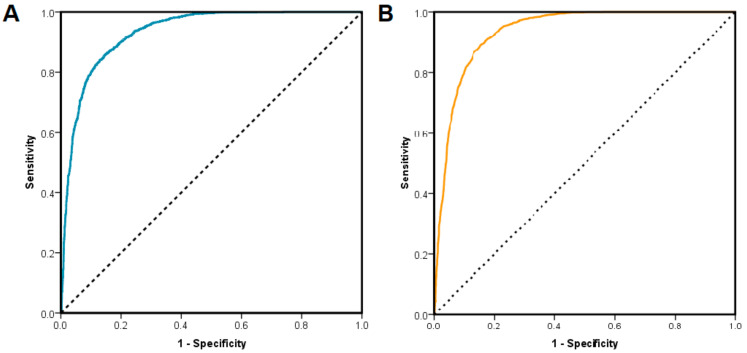
(**A**) ROC curve for classifying PIM-1 inhibitor vs. decoy compounds based on predicted binding energies after docking with AutoDock Vina; (**B**) ROC curve for classifying PIM-1 inhibitors vs. decoy compounds based on predicted binding energies after docking with AutoDock4. Dotted line represents the line of no discrimination (as generated by a random classifier).

A second validation of the docking protocol consisted in evaluating the capacity of the docking algorithms to discriminate between PIM-1 inhibitors and decoys by building ROC (receiver operating characteristic) curves and calculating the ROC AUC (area under receiver operating characteristic curve) values using the predicted binding energies (Figure 7). A ROC AUC value of 0.932 was obtained for AutoDock Vina, and 0.936 for AutoDock4, indicating a good separation between positive (PIM-1 inhibitors) and negative (decoys) ligands based on the molecular docking experiments.

After the virtual screening study using AutoDock vina, the docking results displayed as binding energy values varied overall from −12.77 kcal/mol for the strongest binding inhibitor to −2.77 kcal/mol for the weakest binding decoy compound. Comparative descriptive statistics for binding energy values between the two docked sets can be found in Table 4.

**Figure 8:**
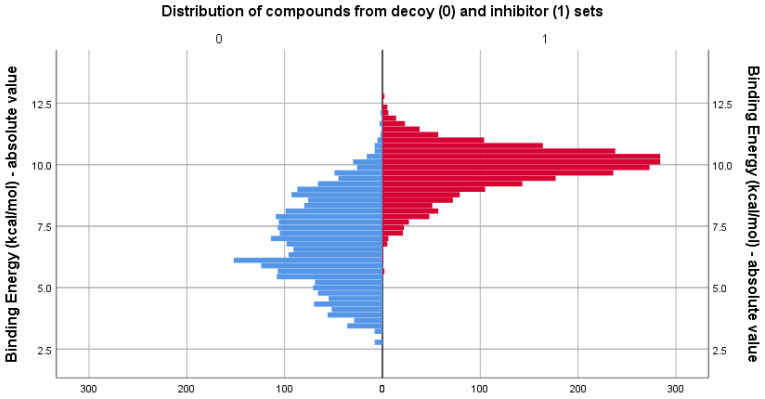
Comparison between the distribution of binding energy, expressed as absolute values, for the decoy (blue) and inhibitor (red) sets, as calculated by Vina algorithm.

As expected, calculated binding affinities were on average lower for the compounds in the decoy set. However, the distribution of the data suggests that some of the compounds in the decoy set are seen as potent inhibitors by the docking algorithm, prompting further investigation and the necessity of docking score correction. The distribution of docking scores expressed as absolute values of the binding energy results for both compared sets is displayed in Figure 8.

### 3.4. Interaction Analysis

After transforming the presence and absence of interactions with all 50 participating amino acid residues of the protein in the docking process, interactions were observed as a fingerprint for each compound, which the current research analyzed to identify patterns that could orient a medicinal chemist to design a compound that interacts with the most efficient residues for potent inhibition activity against the target protein.

Table 5 presents the frequency of interactions between the target protein and ligands from the decoy and inhibitor sets, respectively. Amino acid residues are listed along with the number and percentage of interactions observed in each set. Residues underlined in the table indicate those that predominantly interact with compounds from the decoy set rather than the inhibitor set, representing residues to be avoided in an efficient inhibitor interaction.

The results highlight several key findings. For instance, Ile185 and Val52 exhibited interactions in both the decoy and inhibitor sets, with nearly 100% prevalence in the inhibitor set, as opposed to residues such as Gly47 and Gly48, which had a significantly higher frequency of interactions with compounds from the decoy set compared with the inhibitor set.

Other residues such as Leu174, Phe49, and Asp186 showed a relatively high occurrence of interactions in both sets, suggesting their involvement in binding interactions regardless of the compound type. On the other hand, residues like Pro123 demonstrated a relatively higher proportion of interactions with compounds from the decoy set, indicating their potential specificity towards this set.

These findings provide valuable insights into the differential interactions between the target protein and ligands from the decoy and inhibitor sets. The observed variations in interaction frequencies across residues highlight their potential importance in distinguishing between different types of compounds and can aid in understanding the underlying binding mechanisms.

### 3.5. Binary Logistic Regression Analysis

The binary regression analysis returned several models depending on the classification cutoff value. For each model, the true positive and true negative rates are presented in Table 6 as indicators of the regression performance.

When the regression was performed using the Class 5 variable, the obtained equation contained the binding energy resulting from the docking study and a correction for three amino acids and the water molecule HOH334. The probability of a compound having a pIC_50_ value over 5 is described by the following formula:

$$P_{class5}=\frac{1}{1+e^{-X5}},\;$$

where X5 is

X5 = 0.9228 × BindingEnergy + 0.5394 × Gly45 + 0.3830 × Pro123 + 0.761 × Asp131 + 0.7555 × HOH − 9.8659

The equation indicates that the calculated binding energy was overestimated and the interactions with Gly45, Pro123, and Asp131 were under-evaluated. If a compound has no contact with any of these three amino acids and the water molecule, the binding energy has to be over 10.691 in order to have a pIC_50_ value over 5. If the docking study indicated that a ligand interacted with all four residues, the threshold of the binding energy was 8.048. The sensitivity of the model is 0.809, but it can be increased to 0.90 if the cutoff of the calculated Pclass5 value is lowered to 0.33.

When the regression was performed using the Class 6 variable, the obtained equation contained the binding energy resulting from the docking study and a correction for seven amino acids and the water molecule. This equation is similar to the equation for the P_class5_ value with the addition of the interactions with Ala65, Val126, Glu171, and Asn172. The probability of a compound having a pIC_50_ value over 6 is described by the following formula:

$$P_{class6}=\frac{1}{1+e^{-X6}},\;$$

where X6 is

X6 = 0.7696 × BindingEnergy + 0.6171 × Gly45 + 0.575 × Ala65 + 0.1787 × Pro123 + 0.322 × Val126 + 0.7831 × Asp131 + 0.4953 × Glu171 − 0.2939 × Asn172 + 0.8788 × HOH − 9.9739

This model slightly reduced the number of false positive hits but also reduced the sensitivity to 0.736. The sensitivity can be elevated to 0.90 if the cutoff for the P_class6_ value is lowered to 0.29 with a corresponding specificity of 0.72. Considering that the use of docking studies is mainly for the discovery of new potential inhibitors of PIM-1, the Class 5 cutoff seems to be a better choice to reduce the risk of losing potent inhibitors.

### 3.6. Clustering Analysis of Interaction Data

**Figure 9:**
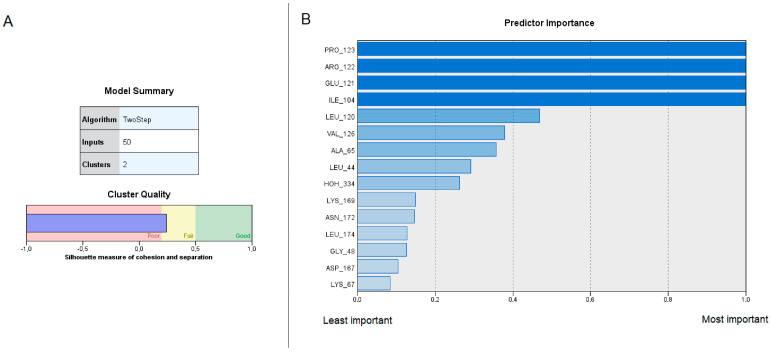
(**A**) Model summary for the two-step clustering analysis of the interactions between the inhibitors and decoy ligands with the participating amino acid residues of the docked protein PIM-1 kinase. Silhouette measure of cohesion and separation indicates a fair classification into the two clusters; (**B**) Importance of top 15 variables in separating cases into the two clusters. The presence of an interaction with residues with greater importance (upper rows) has a higher impact on deciding in which cluster a compound belongs than those with lower importance.

Based on the interaction patterns of each compound with the target protein, the two-step clustering analysis classified the 5080 compounds into two clusters, with the first comprised of 2504 cases (49.3%) and the second of 2576 (50.7%). This corresponds roughly to the two subsets analyzed, decoy compounds and inhibitors, respectively, suggesting the clustering analysis can discriminate well between the two groups. The silhouette measure for the obtained clusters had a value of 0.2392, which suggests that the clustering solution has a moderate level of cohesion and separation, indicating that the cases are somewhat well-matched to their respective cluster and have a reasonable degree of separation from cases in the other cluster. Results are graphically represented in Figure 9A,B. Detailed information about the predictive importance of all 50 interacting residues can be found in Supplementary Materials, Table S1.

From these results we can conclude that residues with higher importance values, such as Ile104, Glu121, Arg122, and Pro123, are key contributors to the clustering solution, having an important role in inhibiting the activity of PIM-1 kinase. Other residues with relatively high importance values, such as Val126, Leu120, Leu44, and Ala65, also exhibit significant relevance to the efficient binding of the protein. A further detailed diagram of the clustering analysis is available in the Supplementary Materials, Figure S1.

### 3.7. Case Study of Predicted Binding Interactions for True Positive, False Positive, False Negative, and True Negative Ligands

**Figure 10:**
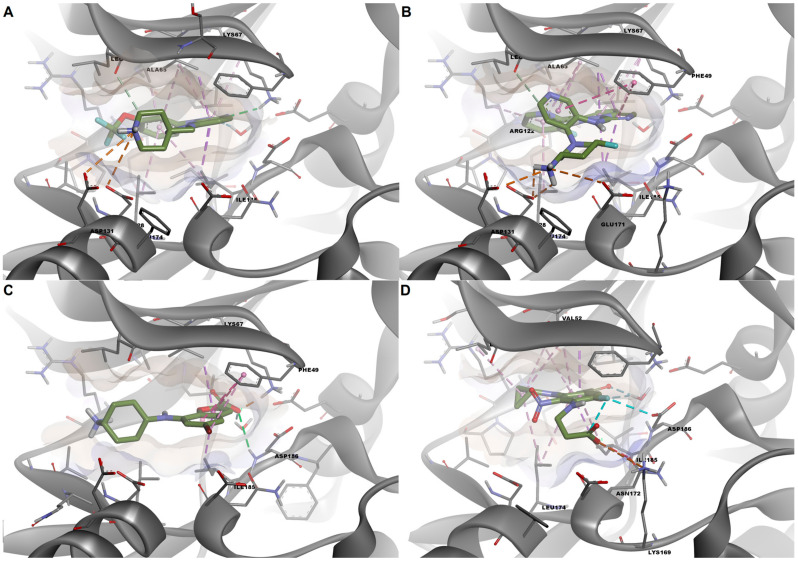
Predicted conformations of selected ligands after docking into PIM-1 active site. (**A**) Predicted conformation of the TP ligand; (**B**) predicted conformation of the FP ligand; (**C**) predicted conformation of the FN ligand; (**D**) predicted conformation of the TN ligand.

**Figure 11:**
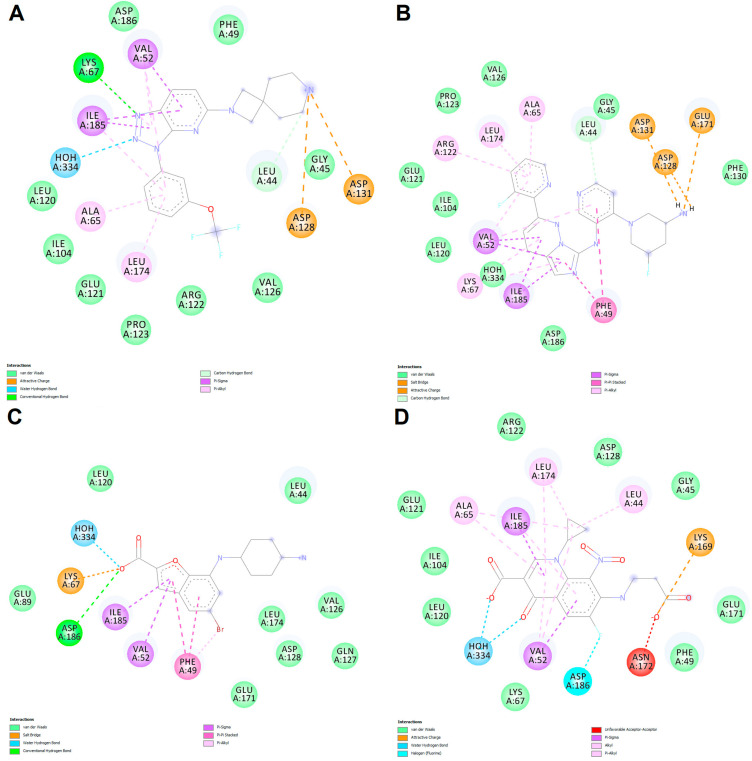
Predicted molecular interactions between selected ligands and PIM-1 kinase. (**A**) Predicted interactions for the TP ligand; (**B**) predicted interactions for the FP ligand; (**C**) predicted interactions for the FN ligand; (**D**) predicted interactions for the TN ligand.

We further chose to discuss the predicted interactions for a set of four selected ligands (Table 7) in order to investigate the binding characteristics that yielded both true and false predictions based on the Class 5 regression model. Firstly, we analyzed the binding mode of a true positive (TP, CHEMBL1952126), which is structurally similar to the co-crystallized inhibitor: the [1,2,4]triazolo[4,3-b]pyridazine scaffold in the co-crystallized inhibitor is replaced by a [1,2,3]triazolo[4,5-b]pyridine scaffold, the cyclohexyl substructure is replaced by an N-(7-azaspiro[3.5]nonan-2-yl)methyl moiety, while the trifluoromethyl-phenyl fragment is replaced by trifluoromethoxy-phenyl. As expected, the true positive ligand interacted with PIM-1 kinase in a similar fashion to the co-crystallized ligand, by forming a hydrogen bond with Lys67, a water hydrogen bond with HOH334, and pi–sigma interactions with Val52 and Ile185. The replacement of the cyclohexane fragment with the N-methylated and positively charged azaspiro[3.5]nonane moiety yielded interactions based on attractive charges with Asp128 and Asp131, while the trifluoromethoxy-phenyl moiety engaged in nonpolar pi–alkyl interactions with Ala65 and Leu174. The TP ligand also made van der Waals contacts with Gly45 and Pro123 (Figure 10A and Figure 11A). Therefore, the TP ligand satisfied the interactions with the three residues and water molecule identified as good predictors via the logistic regression.

Next, another relatively similar compound to the co-crystallized ligand was chosen as an example of a false positive (FP) prediction. The FP ligand (N-[4-[(3S,5R)-3-amino-5-fluoropiperidin-1-yl]pyridin-3-yl]-2-(3-fluoropyridin-2-yl)imidazo[1,5-b]pyridazin-7-amine, CHEMBL4111268) is based on an imidazo[1,5-b]pyridazine scaffold which did not engage in polar interactions with either Lys67 or HOH334, forming only a van der Waals contact with HOH334 and pi–alkyl interactions with Lys67. However, the protonated fluoropyridine–amine moiety formed a salt bridge with Asp131, while the fluoropyridine substructure engaged in pi–alkyl interactions with Ala65 and Leu174. Nonetheless, the same ligand made van der Waals contacts with Gly45 and Pro123 (Figure 10B and Figure 11B). The architecture of this specific compound prevents a favorable orientation into the binding site, thus hindering the formation of polar interactions with Lys67 and the conserved water molecule. However, the high predicted binding affinity (−10.879 kcal/mol) and presence of any form of interactions with the three residues (Gly45, Pro123, Asp131) and HOH334 led to its incorrect classification as a potent PIM-1 inhibitor.

An example of a false negative (FN) result is represented by a PIM-1 kinase inhibitor with an entirely different chemotype (CHEMBL1782530, 7-[(4-aminocyclohexyl)amino]-5-bromo-1-benzofuran-2-carboxylic acid). The carboxylate moiety formed a salt bridge with Lys67 and a water hydrogen bond with HOH334, similar to the α-phosphate of AMP-PNP, and a hydrogen bond with Asp186. Moreover, the benzofuran scaffold formed several nonpolar interactions, such as pi–sigma interactions with Val52 and Ile185, and pi–pi stacked interactions with Phe49. However, the positively charged 4-aminocyclohexyl moiety failed to interact through attractive charges with Asp131 due to an unfavorable orientation of the substructure (Figure 9C and Figure 10C). However, the latter interaction is not essential for PIM-1 kinase inhibitory activity, as revealed by the crystal structure used in this study. The erroneous classification of this particular ligand can be attributed to the lack of contact with Gly45, Pro123, and Asp131, and a relatively low predicted binding affinity (−7.825).

Lastly, we further examined the predicted interactions between PIM-1 and a true negative (TN) ligand, which is also a carboxylic acid derivative (CHEMBL4086292, 7-(2-carboxyethylamino)-1-cyclopropyl-6-fluoro-8-nitro-4-oxoquinoline-3-carboxylic acid). The TN ligand is based on a 4-oxoquinoline scaffold and has two carboxylate moieties. The oxoquinoline derivative formed two water hydrogen bonds with HOH334 but failed to form a salt bridge with Lys67, showing only weak van der Waals contact with this residue. However, the second carboxylate moiety interacted with Lys169 through attractive charges, similar to the γ-phosphate of AMP-PNP, but also exhibited an unfavorable acceptor–acceptor interaction with the same residue. Nonetheless, the main scaffold formed nonpolar interactions with Val52, Ile185, Ala65, and Leu174, similar to other ligands. Considering that the predicted binding energy was higher than the values for the majority of potent inhibitors (−8.362 kcal/mol) and that no contacts were made with Pro123 and Asp131, the ligand was correctly predicted as inactive.

## 4. Discussion

Analyzing the two compound sets in their entirety, the difference in terms of chemical structures between the decoy set and the PIM-1 inhibitors can be easily observed when taking into account chemical descriptors, as the descriptive data suggest that the compounds in the inhibitor set have larger molecular weights, higher lipophilicity, increased potential for hydrogen bonding, larger surface areas, higher complexity and flexibility, and a greater presence of non-carbon atoms and rotatable bonds compared with the compounds in the decoy set. This aspect could be further explored in further similar studies, which could investigate PIM-1 inhibitors compared with other protein kinase inhibitors in order to observe the differences in interaction between the protein kinase inhibitor classes. For the current study, the decoy set was intentionally formed to contain molecules that do not target a specific protein, as this kind of approach is mostly used when training a predictive model, such as for the probability of being active in this case.

An important aspect to cover is the pharmacophore groups of the inhibitors and their impact on the binding affinity towards the protein. The majority of decoy compounds do not possess the pharmacophore groups needed for inducing a stable affinity towards the binding site of the PIM-1 kinase. Several key features, described further, have been identified in the literature by various pharmacophore-based screening studies [21,22,23]. One of the key pharmacophore groups in PIM-1 kinase inhibitors is the hinge-binding motif, which typically consists of a hydrogen bond acceptor and a hydrogen bond donor, needed for interaction with key residues in the kinase’s hinge region. The hydrogen bond acceptor, often an oxygen or nitrogen atom, forms a hydrogen bond with the backbone amino group of the hinge residue, while the hydrogen bond donor, such as an amino or hydroxyl group, interacts with the backbone carbonyl group. These interactions help stabilize the inhibitor within the kinase active site. Indeed, a quick statistical analysis confirms that 2504 out of the 2546 inhibitors (98.35%) possess at least one hydrogen acceptor and one hydrogen group, compared with the decoy molecules with only 1321 structures (52.13%). Additionally, a key feature in PIM-1 kinase inhibitors is the presence of a basic nitrogen atom, which serves as a pharmacophore for interactions with acidic residues in the kinase active site. This salt bridge or electrostatic interaction contributes to the binding affinity and specificity of the inhibitor. In total, 1636 of the inhibitors (64.26%) display at least one basic nitrogen in their molecule, compared to 894 molecules (35.28%) in the decoy group. Another important group is the hydrophobic moiety, which enhances the binding affinity of PIM-1 kinase inhibitors. This hydrophobic group, often an aromatic ring or aliphatic chain, interacts with hydrophobic residues in the kinase binding pocket, contributing to the overall stability of the inhibitor and improving selectivity and potency. As expected, 2545 (99.96%) of the inhibitors possess an aromatic ring in their molecular structure, whereas in the decoy group aromatic rings were present in only 1415 cases (55.84%). Undoubtedly, these aspects are of great importance when identifying or designing a PIM-1 kinase inhibitor, as these features have a direct impact on interaction patterns, conditioning the affinity and stability of the ligand with the active site of the target protein.

In order to observe the manner in which the docking algorithm estimates interactions between the ligands and the targeted protein, we compared the binding energy values of the Autodock4 docking study with the results obtained using the AutoDock Vina algorithm. In terms of discriminating between decoys and inhibitors, data suggest that the two docking algorithms are similar, as indicated by the ROC curve analysis. However, AutoDock4 behaved less successfully in predicting the correct binding pose, also estimating a higher number of interactions with the binding pocket. Therefore, interaction and regression analyses were performed only using the data generated by docking with Vina.

Several binary logistic regression models were trained based on binding energies and interactions with amino acid residues. We further selected a regression equation that used as dependent variables activity classes derived from a pIC_50_ value of 5 M as a threshold, which is a consensus for defining relevant biological activity. The model yielded satisfactory true positive and true negative rates and estimated the probability of PIM-1 inhibitory activity based on binding energy values, and the presence of interactions with three amino acid residues (Gly45, Pro123, and Asp131) and the structural water molecule (HOH334).

In terms of interaction patterns, it can be easily concluded that the two identified clusters correspond to the two binding modalities in which the inhibitors and the decoys interact with the protein. The cluster separation silhouette value suggests that the clustering solution captured meaningful patterns in the data, an aspect that was also highlighted by the regression equation for Class 5 and Class 6, by including amino acid residues with a high contribution in the cluster classification. One notable residue that stands out is Pro123, which exhibits one of the highest predictor importance values. Pro123 is particularly significant as it is part of the hinge region and is specific to PIM-1 kinase, playing a crucial role in disrupting the formation of one of the two hydrogen bonds typically observed between the ATP molecule and the hinge regions of other protein kinases [24]. Asp131 and Gly45, residues also present in Class 5 and 6 equations, appeared to have a lower impact on cluster formation, with predictor importance values of 0.0025 and 0.02, respectively. This could be attributed in part to Gly45’s tendency to form weak van der Waals interactions with most of the ligands (with some exceptions when forming carbon–hydrogen or halogen interactions), and Asp131’s weak salt-bridge interactions with some ligands. In our study, we chose to keep the structural water HOH334, as it can mediate hydrogen bonding between inhibitors and Glu89, a key residue located in the αC-helix. In contrast, other authors chose to delete all the water molecules within the active site prior to performing virtual screening [15]. Overall, the data highlight the variability in residue interactions within the inhibitor set. While some residues show high occurrence and strong interactions, others exhibit lower frequencies, indicating less prominent or less stable interactions with the inhibitors. It is important to consider these variations in residue interactions when analyzing the structural and functional aspects of the protein–ligand interactions, and this aspect is individually applicable to most of the target proteins.

Nonetheless, it is not about a single key interaction with a certain residue, but rather a combination of interactions and lack of interactions with certain amino acid residues that are responsible for good binding of the ligand to the targeted protein, as the current research attempts to explore. High-throughput screening campaigns rely on visual inspection of top-scoring ligands for candidate selection and mostly have success rates of approximately 20% on average after experimental validation [25]. The binding energy can be strongly correlated with the number of heavy atoms and can often prove to be a poor predictor on its own since the establishment of certain molecular interactions with the target binding pocket is essential for the desired activity. Therefore, rescoring of molecular docking results based on predictive models that integrate both docking scores and interactions with key amino acid residues could potentially speed up the selection of promising drug candidates and heighten the success rate for hit discovery.

One possible limitation of the current study is the inclusion of a certain amount of bias in the docking protocol. The optimization steps of the co-crystallized protein–ligand complex prior to docking eventually impact the screening experiment by favoring certain chemotypes which resemble the crystal structure of the known inhibitor. In addition, the regression model could potentially recognize only the predicted strong binders that interact with the binding site in a similar fashion to the ligands used for training. The inclusion of one structural water and optimization of the hydrogen positions could be especially responsible for this outcome. Since the presented docking optimization protocol can be suitable for high-throughput virtual screening, a strategy for overcoming this limitation could be considered. Such strategies could imply repeating the screening on a set number of interesting hit molecules, by performing a more precise docking procedure, using induced-fit (flexible residues) approaches. Following the induced-fit approach, the investigators should visually inspect the binding pose of the ligand and decide whether the outcome warrants a repeated experiment with the exclusion of structural water molecules.

This research suggests current docking methods and virtual screening protocols could be further improved by making use of the widely available data provided by online chemical databases in order to better apply and adjust docking protocols and to interpret the results more deeply, further obtaining richer information about potential hits or leads.

## 5. Conclusions

The current research may be promising for the statistical identification of certain chemical structures for which binding-affinity results obtained from docking studies may be adjusted to better predict the biological activity, depending on which key interacting residues a ligand binds. By identifying and focusing on these key residues, further investigations and experimental studies can be conducted to explore their functional and structural significance in the binding process.

Our findings suggest that these key residues may serve as potential targets for designing compounds with enhanced binding affinity or specificity towards PIM-1 kinase protein. This approach may be further applied to diverse drug targets, possibly improving current knowledge about ligand–protein interaction in certain cases.

## Figures and Tables

**Table 1:** Chemical descriptors used as filter values for decoy set selection (representing range values of the inhibitor set).

Chemical Descriptor	Minimum Value	Maximum Value
Molecular Weight (MW)	162.14	700.11
Total Surface Area (TSA)	178.380	354.020
logP	−1.7191	7.1457
No. of Hydrogen Bond Acceptors (HBA)	2	13
No. of Hydrogen Bond Donors (HBD)	0	8

**Table 2:** Descriptive statistics of chemical descriptors for the inhibitor set (n = 2546).

Descriptor	Minimum	Maximum	Mean	Std. Deviation	Variance
Total_Molweight	162.14	700.11	401.34	74.81	5597.09
cLogP	−1.719	7.146	2.830	1.124	1.263
H_Acceptors	2	13	6.36	1.54	2.36
H_Donors	0	6	2.15	0.91	0.83
Total_Surface_Area	121.65	481.58	290.52	50.35	2534.95
Relative_PSA	0.063	0.639	0.265	0.066	0.004
Molecular_Flexibility	0	0.617	0.323	0.080	0.006
Molecular_Complexity	0.594	1.130	0.885	0.050	0.003
Non_CH_Atoms	3	14	7.79	1.91	3.66
Rotatable_Bonds	0	12	4.03	1.74	3.03

**Table 3:** Descriptive statistics of chemical descriptors for the decoy set (n = 2534).

Descriptor	Minimum	Maximum	Mean	Std. Deviation	Variance
Total_Molweight	162.14	698.84	260.50	87.64	7680.76
cLogP	−1.714	7.091	2.102	1.770	3.135
H_Acceptors	2	13	3.24	1.62	2.62
H_Donors	0	7	0.93	1.11	1.23
Total_Surface_Area	43.46	590.54	192.76	69.67	4853.85
Relative_PSA	−0.053	1.000	0.233	0.143	0.020
Molecular_Flexibility	0	0.913	0.449	0.219	0.048
Molecular_Complexity	0	1.079	0.648	0.172	0.029
Non_C_H_Atoms	2	16	4.00	2.01	4.04
Rotatable_Bonds	0	37	4.05	4.69	21.97

**Table 4:** Descriptive statistics of docking results expressed as binding energy values (−kcal/mol) for the two sets after docking with AutoDock Vina. For easier interpretation, energies have been converted to absolute values.

	Count	Mean	Median	Min.	Max.	Range	Std. Deviation	Variance
Decoy set	2534	−6.8369	−6.8385	−12.0690	−2.7770	9.2920	1.7462	3.0492
Inhibitor set	2546	−9.8245	−9.9535	−12.7760	−5.6140	7.1620	0.9527	0.9076

**Table 5:** Frequency of interactions between the target protein and ligands from the decoy and the inhibitor set, respectively. Underlined residues indicate residues that interact predominantly with compounds from the decoy set rather than the inhibitor set.

Residue	Decoy Set (n = 2534)	Inhibitor Set (n = 2546)	Residue	Decoy Set (n = 2534)	Inhibitor Set (n = 2546)
Ile185	2460 (97.08%)	2546 (100.00%)	Gly47	59 (2.33%)	68 (2.67%)
Val52	2457 (96.96%)	2544 (99.92%)	Gly48	112 (4.42%)	68 (2.67%)
Leu174	2369 (93.49%)	2538 (99.69%)	Asp167	100 (3.95%)	54 (2.12%)
Phe49	2385 (94.12%)	2536 (99.61%)	Ser54	14 (0.55%)	40 (1.57%)
Asp186	2438 (96.21%)	2523 (99.10%)	Ser189	82 (3.24%)	22 (0.86%)
Ala65	2126 (83.90%)	2488 (97.72%)	Pro125	9 (0.36%)	21 (0.82%)
Lys67	2344 (92.50%)	2484 (97.56%)	Glu124	9 (0.36%)	20 (0.79%)
HOH334	2200 (86.82%)	2475 (97.21%)	Asp202	70 (2.76%)	18 (0.71%)
Leu44	1659 (65.47%)	2433 (95.56%)	Thr204	57 (2.25%)	17 (0.67%)
Leu120	2113 (83.39%)	2321 (91.16%)	Glu135	3 (0.12%)	11 (0.43%)
Arg122	1370 (54.06%)	2275 (89.36%)	Gly203	54 (2.13%)	10 (0.39%)
Val126	1030 (40.65%)	2211 (86.84%)	Leu177	3 (0.12%)	7 (0.27%)
Glu171	1490 (58.80%)	2167 (85.11%)	Gly188	56 (2.21%)	7 (0.27%)
Asp128	1165 (45.97%)	2101 (82.52%)	Ser75	24 (0.95%)	5 (0.20%)
Gly45	1100 (43.41%)	2012 (79.03%)	Arg73	0 (0.00%)	2 (0.08%)
Ile104	1868 (73.72%)	1680 (65.99%)	Pro42	0 (0.00%)	1 (0.04%)
Asn172	1539 (60.73%)	1642 (64.49%)	Leu43	0 (0.00%)	1 (0.04%)
Glu121	1304 (51.46%)	1586 (62.29%)	Val69	0 (0.00%)	1 (0.04%)
Pro123	776 (30.62%)	1417 (55.66%)	Ile74	0 (0.00%)	1 (0.04%)
Gln127	358 (14.13%)	1068 (41.95%)	Pro87	0 (0.00%)	1 (0.04%)
Asp131	196 (7.73%)	965 (37.90%)	Arg205	4 (0.16%)	1 (0.04%)
Lys169	628 (24.78%)	836 (32.84%)	Ile66	1 (0.04%)	0 (0.00%)
Phe130	266 (10.50%)	572 (22.47%)	Ile173	1 (0.04%)	0 (0.00%)
Glu89	442 (17.44%)	463 (18.19%)	Lys183	1 (0.04%)	0 (0.00%)
Ser46	190 (7.50%)	430 (16.89%)	Phe187	2 (0.08%)	0 (0.00%)

**Table 6:** The performance of models as a function of the cutoff value.

Dependent Variable	Cox and Snell R Square	True Positive Rate	True Negative Rate	Overall
Class 4	0.543	89.1% (2264/2542)	85.9% (2180/2538)	87.5%
Class 5	0.406	80.9% (1724/2132)	81.4% (2400/2948)	81.2%
Class 6	0.364	73.6 % (1343/1824)	82.2% (2678/3256)	79.2%
Class 7	0.305	56.4% (771/1366)	87.5% (3248/3714)	79.1%

**Table 7:** ChEMBL IDs, experimental potencies, and predicted variables for the selected ligands.

Category	ChEMBL ID	pIC_50_ (M)	Binding Energy (kcal/mol)	P (Class 5)	Contacts at All Four Sites
TP	CHEMBL1952126	8.398	−10.751	0.9237	yes
FP	CHEMBL4111268	4.357	−10.879	0.9316	yes
FN	CHEMBL1782530	9.000	−7.825	0.1313	no
TN	CHEMBL4086292	1.261	−8.362	0.2985	no

TP—true positive; FP—false positive; FN—false negative; TN—true negative; P—probability of being active.

## Data Availability

Data regarding ChEMBL IDs of the 5080 compounds and their respective docking results, interaction coding, and logistic regression model results can be found in Supplementary File S2.

## Supplementary Materials

The following supporting information can be downloaded at: https://www.mdpi.com/article/10.3390/life13081635/s1, Table S1: Predictor importance for all 50 binary variables representing the participating amino acid residues of the protein and their interaction with a ligand. Values range between 0 and 1, with 1 representing the highest importance, meaning the presence of an interaction with the importance of 1 has a very high impact on the clustering solution, while the interaction or lack of it with a residue with predictor importance close to 0 does not very much affect the placement of a case into one cluster or the other. Figure S1: Clustering analysis model view of cluster cells, as generated by SPSS two-step clustering classification analysis, with cluster center value (left) and relative distributions (right) for the binary value of the respective cell. The table is continued in the next two pages, with a repetition of the last row for easier reading.
