# Supplementary material for: A PIM-1 Kinase Inhibitor Docking Optimization Study Based on Logistic Regression Models and Interaction Analysis
Source: Life (Basel). 2023 Jul 27;13(8):1635. doi: 10.3390/life13081635 (PMC10455354; doi:10.3390/life13081635)

life

Article

A PIM-1 Kinase Inhibitor Docking Optimization Study Based on Logistic Regression Models and Interaction Analysis

George Nicolae Daniel Ion, George Mihai Nitulescu * and Dragos Paul Mihai

Faculty of Pharmacy, "Carol Davila" University of Medicine and Pharmacy, Traian Vuia 6, 020956 Bucharest, Romania; daniel.ion@drd.umfcd.ro (G.N.D.I.); dragos_mihai@umfcd.ro (D.P.M.)
* Correspondence: george.nitulescu@umfcd.ro

Supplementary material

Table S1. Predictor importance for all 50 binary variables representing the participating amino acid residues of the protein and their interaction with a ligand. Values range between 0 and 1, with 1 representing the highest importance, meaning the presence or absence of an interaction with the importance of 1 has a very high impact on the clustering solution, while the interaction or lack of it with a residue with predictor importance close to 0 doesn't affect very much the placement of a case into one cluster or the other.

Variable	Predictor importance	Variable	Predictor importance
ILE_104	1	SER_75	0.0227
GLU_121	1	GLU_171	0.021
ARG_122	1	GLY_45	0.02
PRO_123	1	SER_46	0.0152
LEU_120	0.469	ASP_186	0.0127
VAL_126	0.3793	GLU_135	0.0091
ALA_65	0.3567	PHE_130	0.0059
LEU_44	0.2907	ARG_205	0.0051
HOH_334	0.2628	LEU_177	0.0039
LYS_169	0.1495	ARG_73	0.0026

Citation: Ion, G.N.D.; Nitulescu, G.M.; Mihai, D.P. A PIM-1 Kinase Inhibitor Docking Optimization Study Based on Logistic Regression Models and Interaction Analysis. *Life* **2023**, *13*, x. https://doi.org/10.3390/life13081635

Academic Editor: Yong Weon Yi

Received: 27 May 2023
Revised: 16 July 2023
Accepted: 25 July 2023
Published: 27 July 2023

Copyright: © 2023 by the authors. Submitted for possible open access publication under the terms and conditions of the Creative Commons Attribution (CC BY) license (https://creativecommons.org/licenses/by/4.0/).

ASN_172	0.1466	ASP_131	0.0025
LEU_174	0.1268	PRO_42	0.0017
GLY_48	0.1263	ILE_66	0.0017
ASP_167	0.1043	LYS_183	0.0017
LYS_67	0.0834	PRO_125	0.0017
SER_189	0.0764	LEU_43	0.0016
GLY_47	0.0666	VAL_69	0.0016
ASP_202	0.0649	ILE_74	0.0016
VAL_52	0.0585	PRO_87	0.0016
ILE_185	0.0549	ILE_173	0.0016
THR_204	0.0521	GLU_124	0.0013
GLY_203	0.0477	ASP_128	0.0005
GLY_188	0.047	GLU_89	0.0002
PHE_49	0.0289	SER_54	0.0001
GLN_127	0.023	PHE_187	0.0000

Figure S1. Clustering analysis model view of cluster cells, as generated by SPSS Two-step clustering classification analysis, with cluster center value (left) and relative distributions (right) for the binary value for the respective cell. The table is continued in the next two pages, with a repetition of the last row for easier reading.

Clusters

Input (Predictor) Importance

1,0 0,8 0,6 0,4 0,2 0,0

Cluster	2	1
Label		
Description		
Size	50,7% (2576)	49,3% (2504)
Inputs	ARG_122 0 (55,7%)	ARG_122 1 (100,0%)
	GLU_121 0 (82,8%)	GLU_121 1 (97,8%)
	ILE_104 0 (55,4%)	ILE_104 1 (95,8%)
	PRO_123 0 (95,3%)	PRO_123 1 (82,7%)
	LEU_120 1 (75,5%)	LEU_120 1 (99,4%)
	VAL_126 0 (51,5%)	VAL_126 1 (79,6%)
	ALA_65 1 (81,9%)	ALA_65 1 (100,0%)
	LEU_44 1 (69,5%)	LEU_44 1 (91,9%)
	HOH_334 1 (84,9%)	HOH_334 1 (99,4%)
	LYS_169 0 (62,2%)	LYS_169 0 (80,4%)
	ASN_172 1 (72,1%)	ASN_172 1 (52,8%)
	LEU_174 1 (93,3%)	LEU_174 1 (100,0%)

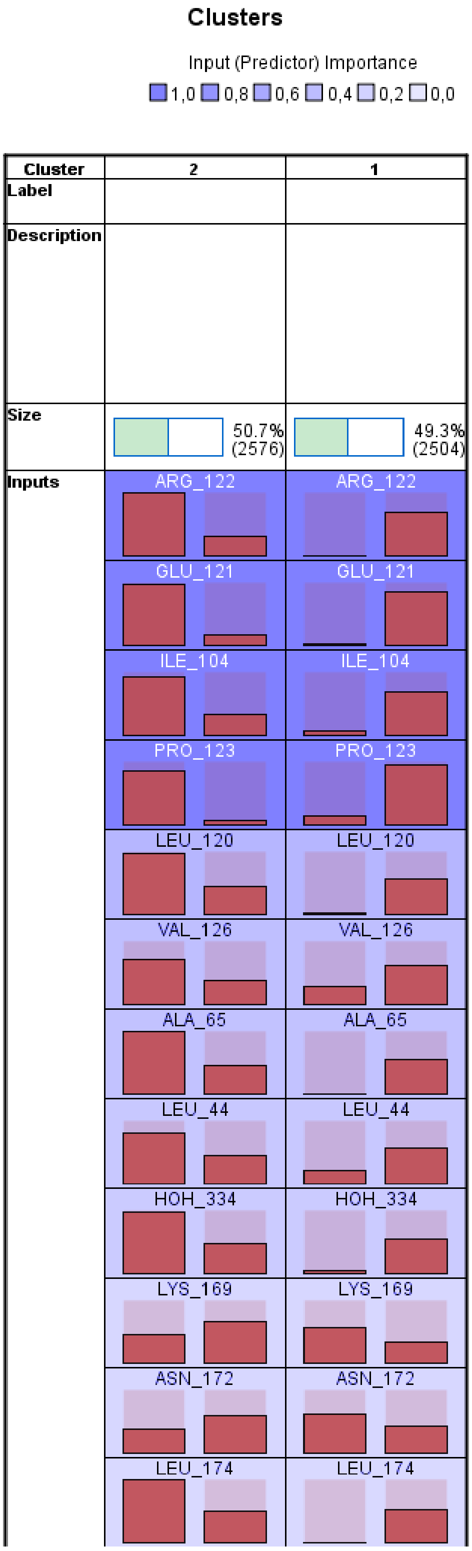

LEU_174 1 (93,3%)	LEU_174 1 (100,0%)
GLY_48 0 (93,1%)	GLY_48 0 (99,9%)
ASP_167 0 (94,1%)	ASP_167 0 (99,9%)
LYS_67 1 (91,8%)	LYS_67 1 (98,3%)
SER_189 0 (96,0%)	SER_189 0 (100,0%)
GLY_47 0 (95,5%)	GLY_47 0 (99,6%)
ASP_202 0 (96,6%)	ASP_202 0 (100,0%)
VAL_52 1 (96,9%)	VAL_52 1 (100,0%)
ILE_185 1 (97,1%)	ILE_185 1 (100,0%)
THR_204 0 (97,2%)	THR_204 0 (100,0%)
GLY_203 0 (97,5%)	GLY_203 0 (100,0%)
GLY_188 0 (97,6%)	GLY_188 0 (100,0%)
PHE_49 1 (98,3%)	PHE_49 1 (95,4%)
GLN_127 0 (68,6%)	GLN_127 0 (75,4%)
SER_75 0 (98,9%)	SER_75 0 (100,0%)
GLU_171 1 (75,2%)	GLU_171 1 (68,7%)
GLY_45 1 (57,9%)	GLY_45 1 (64,7%)
SER_46 0 (85,9%)	SER_46 0 (89,8%)
ASP_186 1 (96,9%)	ASP_186 1 (98,5%)
GLU_135 0 (99,5%)	GLU_135 0 (100,0%)

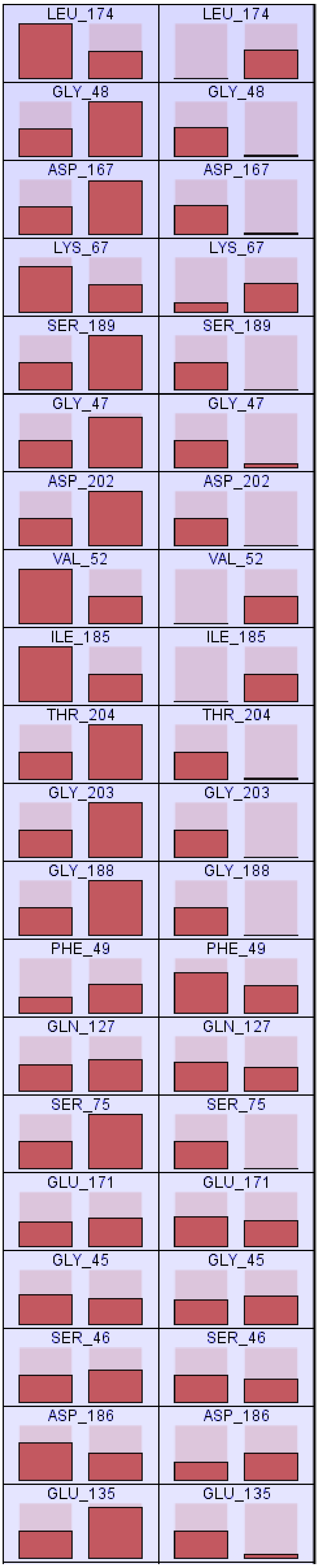

	GLU_135<br>0 (99,5%)	GLU_135<br>0 (100,0%)
	PHE_130<br>0 (82,3%)	PHE_130<br>0 (84,8%)
	ARG_205<br>0 (99,8%)	ARG_205<br>0 (100,0%)
	LEU_177<br>0 (99,7%)	LEU_177<br>0 (99,9%)
	ARG_73<br>0 (99,9%)	ARG_73<br>0 (100,0%)
	ASP_131<br>0 (78,0%)	ASP_131<br>0 (76,3%)
	PRO_125<br>0 (99,3%)	PRO_125<br>0 (99,5%)
	ILE_66<br>0 (100,0%)	ILE_66<br>0 (100,0%)
	LYS_183<br>0 (100,0%)	LYS_183<br>0 (100,0%)
	PRO_42<br>0 (100,0%)	PRO_42<br>0 (100,0%)
	ILE_173<br>0 (100,0%)	ILE_173<br>0 (100,0%)
	ILE_74<br>0 (100,0%)	ILE_74<br>0 (100,0%)
	LEU_43<br>0 (100,0%)	LEU_43<br>0 (100,0%)
	PRO_87<br>0 (100,0%)	PRO_87<br>0 (100,0%)
	VAL_69<br>0 (100,0%)	VAL_69<br>0 (100,0%)
	GLU_124<br>0 (99,3%)	GLU_124<br>0 (99,5%)
	ASP_128<br>1 (64,1%)	ASP_128<br>1 (64,5%)
	GLU_89<br>0 (82,1%)	GLU_89<br>0 (82,3%)
	SER_54<br>0 (99,0%)	SER_54<br>0 (98,9%)
	PHE_187<br>0 (100,0%)	PHE_187<br>0 (100,0%)

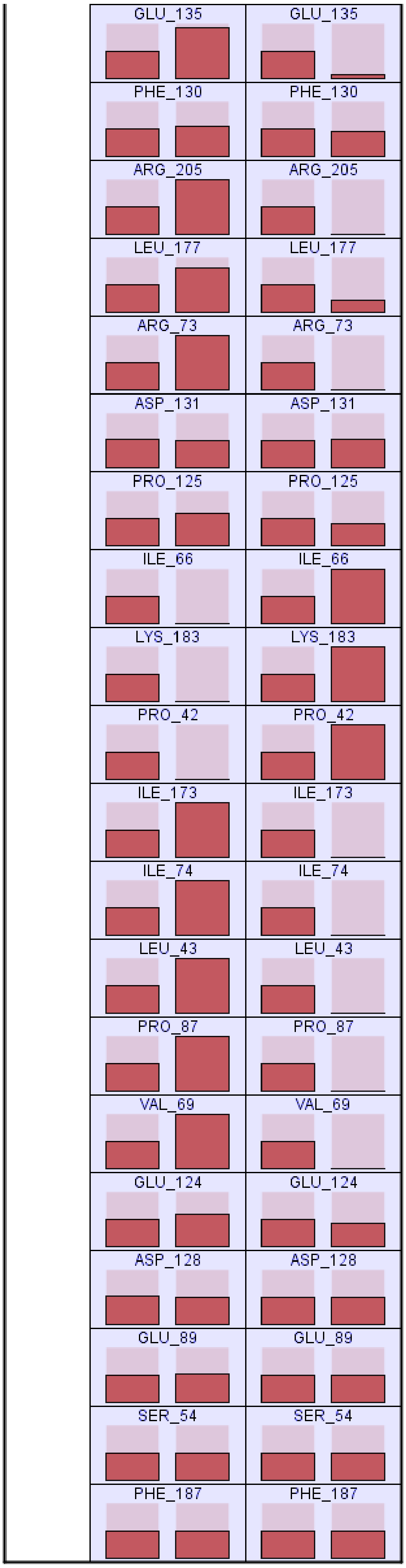

Supplement: Supplementary file 1 [file life-13-01635-s001.zip › Supplimentary File S1.pdf]
